# “Next top” mouse models advancing CTCL research

**DOI:** 10.3389/fcell.2024.1372881

**Published:** 2024-04-10

**Authors:** Yixin Luo, Frank R. de Gruijl, Maarten H. Vermeer, Cornelis P. Tensen

**Affiliations:** Department of Dermatology, Leiden University Medical Center, Leiden, Netherlands

**Keywords:** CTCL (cutaneous T-cell lymphoma), genetically engineered mouse model (GEMM), syngeneic transplantation, xenograft transplantation, Sézary syndrome (SS), mycosis fungoides (MF)

## Abstract

This review systematically describes the application of *in vivo* mouse models in studying cutaneous T-cell lymphoma (CTCL), a complex hematological neoplasm. It highlights the diverse research approaches essential for understanding CTCL’s intricate pathogenesis and evaluating potential treatments. The review categorizes various mouse models, including xenograft, syngeneic transplantation, and genetically engineered mouse models (GEMMs), emphasizing their contributions to understanding tumor-host interactions, gene functions, and studies on drug efficacy in CTCL. It acknowledges the limitations of these models, particularly in fully replicating human immune responses and early stages of CTCL. The review also highlights novel developments focusing on the potential of skin-targeted GEMMs in studying natural skin lymphoma progression and interactions with the immune system from onset. In conclusion, a balanced understanding of these models’ strengths and weaknesses are essential for accelerating the deciphering of CTCL pathogenesis and developing treatment methods. The GEMMs engineered to target specifically skin-homing CD4^+^ T cells can be the next top mouse models that pave the way for exploring the effects of CTCL-related genes.

## 1 Introduction

Cutaneous T-cell lymphoma (CTCL), a rare form of non-Hodgkin lymphoma comprising about 3% of cases, presents unique challenges in oncological research. Characterized by malignant T-cell accumulation in the skin, often without initial spread beyond this organ, CTCL exemplifies the complexity and variability of rare hematological malignancies (Willemze et al., 2018). CTCL represents a heterogeneous group of disorders, including subtypes such as mycosis fungoides (MF), Sézary syndrome (SS) and CD30^+^ lymphoproliferative disorders (LPDs). While primarily a skin disease, CTCL can evolve into systemic lymphoma, spreading to lymph nodes and internal organs. As it constitutes approximately 75% of all primary cutaneous lymphomas, understanding CTCL’s intricate pathobiology demands comprehensive and detailed research approaches (Tensen et al., 2022). In this regard, *in vivo* mouse models are potentially powerful tools in unraveling the complexities of CTCL’s pathogenesis. Such a model can subsequently lead to the design matching well-targeted (early stage) treatments which can then be preclinically tested in this experimentally accessible model.

This review will examine a range of *in vivo* models used in research on CTCL, focusing on various transplantation models and genetically engineered mouse models (GEMMs). We will clarify the subtypes of CTCL if the references classify the model clearly. When the model’s CTCL subtype is not clear, we will use the term ‘CTCL model’ broadly to encompass the diverse spectrum of this disease. Each model type provides insights into different aspects of the disease, from the interaction between tumor cells and the host environment and gene functions to the validation of drug efficacy. These contribute to our deepening understanding of CTCL and aid in the advancement of innovative therapeutic approaches. Here we will categorize and introduce mouse models of CTCL including the next top mouse models, offering a reference for researchers unfamiliar with the field of mouse experimentation when selecting mouse models as a vehicle for CTCL research.

## 2 Transplantation mouse models in CTCL research

Transplant models are essential in CTCL research and typically involve transplanting (human) donor cells or tissues into a recipient organism (mice). CTCL transplant models focused on late-stage human cutaneous lymphomas. These ‘Xenograft models’ entail transplants between different species, by necessity involving immunodeficient mice receiving human CTCL cells or tissues. However, these models lack a fully competent immune system, which is a significant limitation, as it prevents a complete understanding of immune system interactions in CTCL. Additionally, they primarily focus on established tumors, offering limited insight into the early stages of CTCL pathogenesis. This underscores the need for cautious interpretation of results from these models, especially regarding immune response and early disease development. ‘Syngeneic transplant models’ use genetically identical mice to avoid graft-host reactions and preserve normal interactions between tumor and immune system, but do not involve genuine human CTCL. These models, detailed in subsequent sections, offer valuable insights into CTCL pathogenesis and treatment response (Voskoglou-Nomikos et al., 2003).

### 2.1 Immunodeficient mouse models with transplantation

Immunodeficient mouse models are critical for CTCL research, allowing the study of tumor progression and response to treatments. These models are particularly valuable for precision medicine, facilitating individualized testing of medication in the laboratory to address disease heterogeneity. Patient-derived xenograft (PDX) and cell line-derived xenograft (CDX) models, wherein tumor cells from patients or established cell lines from MF, SS and other CTCL subtypes are transplanted into immunodeficient mice, play a key role. Among the cell lines utilized to study CTCL, SeAx, Sez4, SZ4, H9, and Hut78 correspond to SS origin, providing insight into this subtype. Similarly, Myla and HH cell lines reflect advanced MF, while Mac2A and PB2B are indicative of CD30^+^ LPDs, and MJ and Hut102 lines are associated with Adult T-cell Leukemia/Lymphoma (ATLL), demonstrating the broad spectrum of CTCL manifestations (Gill et al., 2022). The recipient mice for the PDX and CDX model, due to targeted genetic modifications that eliminate certain crucial immune functions, do not reject the transplanted cells or samples (Mosier, 1990). However, the absence of a fully functional tumor microenvironment and a comprehensive host immune response are notable limitations of these models.

Current CTCL research lacks comprehensive studies comparing engraftment efficiency and metastatic rates in various immunodeficient mouse strains (Shultz et al., 2005; Cortes et al., 2021; Yamashita et al., 2021). Predominantly, NSG, NOG, and NRG mice have been preferred in recent CTCL studies due to their superior engraftment capabilities, particularly effective in researching human acute leukemia and melanoma (Agliano et al., 2008; Carreno et al., 2009). Under specific pathogen-free conditions, these strains exhibit longer lifespans, enhancing their value in xenotransplantation. The pioneering nude mouse model, despite its historical significance in cancer research, shows lower engraftment success (Shultz et al., 2005).

Other strains like NSB, C.B-17 SCID Beige, and Rag2 −/− γ −/− mice, despite shorter lifespans, display impressive engraftment abilities. Each strain offers unique traits beneficial for specific research purposes. For instance, NOD SCID mice are crucial in studying CTCL’s pruritic phenotype (Prochazkal et al., 1992; Brehm et al., 2013).

Selecting the right immunodeficient mouse strain is critical for CTCL research and depends on study goals, graft nature, and experimental conditions. Thoughtful selection is key to translating preclinical results into clinical applications and advancing CTCL understanding and treatment. Below we outline and compare various immunodeficient mice used in CTCL research (refer to Table 1; Table 2).

**TABLE 1:** The main features of immunodeficient mouse models for transplantation in CTCL research.

Mouse strain	Mutated gene	Cell population change	Main features
NSG Shultz et al. (2005)	Prkdc^scid^ Il2rg^tm1Wjl^	No T cells	Long lifespan: a median survival time of 89 weeks
		No B cells	High engraftment ability
		No NK cells	
NOG Yamashita et al. (2021)	Prkdc^scid^ Il2rg^tm1Sug^	No T cells	Similar to NSG
		No B cells	Long lifespan
		No NK cells	High engraftment ability
NRG Pearson et al. (2008)	Rag1^tm1Mom^	No T cells	Similar to NSG
	Il2rg^tm1Wjl^	No B cells	Long lifespan
		No NK cells	High engraftment ability
NSB Agliano et al. (2008)	Prkdc^scid^ B2m^tm1Unc/J^	No functional T cells	Short lifespan: the average lifespan 30 weeks. High engraftment ability
		No functional B cells	
		Diminished NK cells	
		No MHC class I	
		No complement factor C5	
Nude nu/nu Kaushik et al. (1995)	Foxn1^nu^	No T cells	Moderate lifespan: 6 months to 1 year
			Low engraftment ability
NOD SCID Brehm et al. (2013)	Prkdc^scid^ (NOD background)	No functional T cells	Short lifespan: the median survival time: 37 weeks
		No functional B cells	Moderate engraftment ability. Slightly lower than NSG, NOG and NRG
CB 17 SCID Charley et al. (1990)	Prkdc^scid^ (C.B-17 background)	No T cells	Moderate lifespan: around 1 year
		No B cells	Moderate engraftment ability
CB17 SCID beige Shibata et al. (1997)	SCID beige	No T cells	Moderate lifespan
		No B cells	High engraftment ability
		Defective NK cells	
Rag2 −/− γc−/−Chicha et al. (2005)	Rag2^tm1.1Flv^	No T cells	Short lifespan: the average lifespan 34 weeks. High engraftment ability
	Il2rg^tm1.1Flv^	No B cells	
		No NK cells	

**TABLE 2:** Transplantation methods for immunodeficient mouse models in CTCL research.

Mouse strain	Subcutaneous injection	Intrahepatreal injection	Intrafemoral injection	Intravenous injection	Skin/tumor grafting
NSG Shultz et al. (200)	- SS Blood-derived PBMCs: 1 × 10^6^ cells Poglio et al. (2021)	- Cell lines: Myla (MF cell line), Hut78 (SS cell line), HH (MF cell line): 5 × 10^6^ cells Andrique et al. (2016)	- SS Blood-derived PBMCs: 1 × 10^6^ cells Poglio et al. (2021)	- SS Blood-derived PBMCs: (5–20) × 10^6^ cells Habault et al. (2022) -Cell lines: Hut102 (ATLL cell line): 10 × 10^6^ cells Wang et al. (2020b); Wang et al. (2023) HH (MF cell line), transduced with lentivirus to express click beetle luciferase green and green fluorescent protein and were further transduced to express truncated CD19 (HH-CBG-GFP-t19) Watanabe et al. (2023) HH (MF cell line) (HH-CBR-GFP): 0.5 × 10^6^ Xiang et al. (2023)	
	- MF lymph node-derived leukocytes: (5–10) × 10^6^ cells Wu et al. (2021)				
	- PDX mouse tumor-derived: 4 × 10^6^ cells Wu et al. (2022)				
	- Cell lines: Myla (MF cell line): 0.8 × 10^6^ cells, transduced with miR-125b-5p Manfe et al. (2013)				
	Hut78 (SS cell line) or HH (MF cell line): 1 × 10^6^ cells, transduced with TOX-shRNA Huang et al. (2015)				
	Hut78 (SS cell line) or Myla (MF cell line): 2.5 × 10^6^ cells Jain et al. (2015)				
	Hut78 (SS cell line) or HH (MF cell line): 1 × 10^6^ cells Wu et al. (2015)				
	HH (MF cell line): 2 × 10^6^ cells Jain et al. (2017)				
	Myla (MF cell line), PB2B (CD30^+^ LPDs cell line), HH (MF cell line), H9 (SS cell line), Hut78 (SS cell line), SZ4 (SS cell line), MJ (ATLL cell line), or Hut102 (ATLL cell line): 2.5 × 10^6^ cells Netchiporouk et al. (2017)				
	Hut78 (SS cell line), H9 (SS cell line), MJ (ATLL cell line), or HH (MF cell line): 10 × 10^6^ Wu et al. (2021)				
	Hut78 (SS cell line) or HH (MF cell line): 5 × 10^6^ cells Zhang et al. (2018), Lin et al. (2021)				
	SeAx (SS cell line): 0.4 × 10^6^ cells (ear) Gallardo et al. (2018)				
	SeAx (SS cell line): 3 × 10^6^ cells (flank) Froehlich et al. (2019)				
	MJ (ATLL cell line): 3.8 × 10^6^ cells/sites, transduced with INSL3-shRNA-TRC58, -TRC61 Velatooru et al. (2023)				
NOG Yamashita et al. (2021)	- Cell lines	**-**	-	-	-
	HH (MF cell line): 10 × 10^6^ cells Ito et al. (2009)				
	Myla (MF cell line): 0.2 × 10^6^ cells; HH (MF cell line): 2 × 10^6^ cells; Hut78 (SS cell line): 4 × 10^6^ cells Kitadate et al. (2016)				
	Myla (MF cell line): 1 × 10^6^ cells Matsuda et al. (2022)				
	Myla (MF cell line) or HH (MF cell line): 0.2 × 10^6^ cells, transfected with GFP-miR-150 Ito et al. (2014), Abe et al. (2017)				
NRG Pearson et al. (2008)	- Cell lines	-	-	- Cell lines	-
	Luciferized Hut78 (SS cell line): 2 × 10^6^ or 4 × 10^6^ cells Cortes et al. (2021)			CD38 knockout luciferized H9 (SS cell line) Isabelle et al. (2022)	
NSB Agliano et al. (2008)	**-** Cell lines	**-**	-	-	-
	Myla 2059 (MF cell line): 1 × 10^6^ cells Krejsgaard et al. (2010), Thode et al. (2015)				
	Myla 2000 (MF cell line): 1 × 10^6^ Pedersen et al. (2013)				
Nude nu/nu Kaushik et al. (1995)	**-** Cell lines	**-**	-	-	- Myla-derived mouse tumor fragments: 5 × 3 mm subcutaneously. Thaler et al. (2004)
	Myla (MF cell line): 10 × 10^6^ cells Thaler et al. (2004)				
	HH (MF cell line): 20 × 10^6^ cells Nakahashi et al. (2022)				
	HH (MF cell line): 10 × 10^6^ cells Bresin et al. (2020), Wang et al. (2020)				
	HH (MF cell line): 2 × 10^6^ cells Le et al. (2018)				
NOD SCID Brehm et al. (2013)	- Cell lines	-	-	- Cell lines: Hut102 (ATLL cell line): 10 × 10^6^ cells with sublethally irradiated (1.8 Gy) Yu et al. (2021)	
	Myla (MF cell line): 10 × 10^6^ cells Chen et al. (2023), Furutani et al. (2023)				
	Hut102 (ATLL cell line): 2 × 10^6^ cells Yu et al. (2021)				
	HH (MF cell line): 20 × 10^6^ cells, transduced with PAK1-sh2-vector Wang et al. (2018)				
	HH (MF cell line): 5 × 10^6^ cells, transduced with BIN1- siRNA, Esmailzadeh et al. (2015)				
	SeAx (SS cell line): 0.2 × 10^6^ cells Kato et al. (2016)				
CB 17 SCID Charley et al. (1990)	- Cell lines: HH (MF cell line): 5 × 10^6^ cells Tonozuka et al. (2023)				- Skin grafting from SS: 4 × 7 × 7.5 mm into 4 pieces. Charley et al. (1990)
CB17 SCID beige Shibata et al. (1997)	- Cell lines	-	-	-	-
	Hut78 (SS cell line): 3 × 10^6^ cells Doebbeling (2010)				
	Myla 2059 (MF cell line): 3 × 10^6^ cells Doebbeling (2010)				
	HH (MF cell line): 1 × 10^6^ Nakajima et al. (2019)				
Rag2 −/− γc−/−Chicha et al. (2005)	-	- Cell lines: Hut78 (SS cell line) or SeAx (SS cell line): (0.5–6) × 10^6^ cells van der Fits et al. (2012)	-	- SS Blood-derived PBMCs: (0.5–1) × 10^6^ cells (with irradiation) Gao et al. (2023)	

CTCL: cutaneous T cell lymphoma; PBMC: peripheral blood mono-nuclear cell; SS: Sézary syndrome; MF: mycosis fungoides.

#### 2.1.1 NSG mouse in CTCL

NSG mouse strain, formally named NOD. Cg-Prkdc^scid^ Il2rg^tm1Wjl^, is indispensable for CTCL studies because of its broad immunodeficiency (Shultz et al., 2005). Their unique genetic background amalgamates traits from NOD, SCID (Prkdc^scid^), and gamma mutation (Il2rg^tm1Wjl^), resulting in the absence of functional T cells, B cells, and NK cells. The profound immunodeficiency of NSG mice positions them as an exemplary recipient for development of intrahepatic xenograft models of CTCL, facilitating the evaluation of tumorigenicity and therapeutic responses. The maintenance of NSG mice requires stringent pathogen-free conditions due to their lack of immune defenses, which has implications for the management and costs of these studies. Despite this, the NSG model’s inability to mount an adaptive immune response offers an excellent recipient.

With the aid of this model, researchers have progressively unveiled tumor-driving pathways and corresponding treatment of CTCL (Manfe et al., 2013; Huang et al., 2015; Jain et al., 2015; Wu et al., 2015). Furthermore, the NSG mouse model has demonstrated its utility in the rapid assessment of CTCL (Andrique et al., 2016), and in subsequently testing novel therapeutic modalities. (Jain et al., 2017). These studies underscore the importance of CTCL heterogeneity and highlight the therapeutic potential of coordinated treatments (Netchiporouk et al., 2017; Gallardo et al., 2018; Zhang et al., 2018; Froehlich et al., 2019; Wang et al., 2020; Poglio et al., 2021; Wu et al., 2021). Recent advancements include the superior performance of CCR4-IL2 immunotoxin (Wang et al., 2023), RT39 peptide therapy (Habault et al., 2022), novel drug NT1721 (Lin et al., 2021), JAK3-INSL3 fusion transcripts (Velatooru et al., 2023), anti-CCR4 CAR T cells (Watanabe et al., 2023), universal CD2 CAR-T therapy (Xiang et al., 2023) and the antibody-drug conjugate SGN-CD70A (Wu et al., 2022) have further expanded the therapeutic research landscape for CTCL.

#### 2.1.2 NOG mouse in CTCL

The NOG mouse model, formally designated as NOD. Cg-Prkdc^scid^ Il2rg^tm1Sug^, stands out in CTCL research for its pronounced immunodeficiency, miming severe combined immunodeficiency (SCID) in humans (Ohbo et al., 1996). In close similarity to NSG, this strain is void of functional B and T lymphocytes due to the *Prkdc* ^*scid*^ mutation and lacks natural killer (NK) cells due to the IL-2Rγ^null^ mutation, making them an ideal platform for human cell engraftment (Ito et al., 2009). The NOG model has revealed the tumor-suppressive role of microRNA-16, while IL-22 may facilitate tumor metastasis (Kitadate et al., 2016; Matsuda et al., 2022). Furthermore, miR-150 has demonstrated potential in inhibiting tumor metastasis (Ito et al., 2014) and histone deacetylase inhibitors targeting miR-150 and CCR6, such as Vorinostat, have presented new strategies for the treatment of advanced CTCL (Abe et al., 2017).

#### 2.1.3 NRG mouse in CTCL

The official name for the NRG mouse model is NOD. Cg-Rag1^tm1Mom^ Il2rg^tm1Wjl^. Due to the knockout of the Rag1 and Il2rg genes, this mouse model lacks mature T, B, and NK cells (Pearson et al., 2008). The studies using NRG mice for CTCL research found that the combined use of chlorpromazine and romidepsin displayed significant antitumor activity (Cortes et al., 2021), and the expression of CD38 is associated with the progression of CTCL, suggesting that CD38 could potentially become a new target for therapeutic intervention (Isabelle et al., 2022).

#### 2.1.4 NSB mouse in CTCL

The NSB mouse model with the official name NOD. Cg-Prkdc ^scid^ B2m ^tmUnc/J^, distinct in their immunodeficiency due to a B2m^tm1Unc^ mutation affecting MHC class I expression, lack CD8^+^ T cells and exhibit impaired NK cell function (Christianson et al., 1997). This characteristic enables the strain to support the engraftment of malignant T cells such as Myla 2059, providing a robust model for the study of late-stage CTCL, especially MF, dissemination and treatment (Krejsgaard et al., 2010; Pedersen et al., 2013). Additionally, the secretion of molecules such as galectin-1 and -3 by malignant T cells has been associated with the disruption of skin architecture and the proliferation of keratinocytes in CTCL (Thode et al., 2015).

Distinct from the NOG and NSG strains, which suffer from impaired NK cell function due to mutations in the IL2R gamma chain, the deficit in this strain arises from the impact of the B2m mutation on MHC class I expression, marking its unique role in the study of CTCL models.

#### 2.1.5 Nude (nu/nu) mouse in CTCL

The “nude” (nu/nu) mouse model, which lacks a mature thymus due to a Foxn1 gene mutation, resulting in underdeveloped T cells (Kaushik et al., 1995), has become a critical model for evaluating CTCL therapies, especially in terms of treatment responses for MF patient-derived skin lesions. These mice with transplants of MF have shown superior responses to combination therapies like PUVA and mogamulizumab, a monoclonal antibody targeting CCR4, compared to monotherapies (Thaler et al., 2004; Nakahashi et al., 2022). Studies using “nude” mice highlight the potential of Vorinostat and the HIF-1α inhibitor Echinomycin in CTCL management (Wang et al., 2020; Xia et al., 2020). The dual PI3K/mTOR inhibitor PF-502 extends survival, suggesting its promise in treating CTCL (Bresin et al., 2020). Additionally, metabolic analysis of CTCL-transplanted mice has identified changes in L-glutamate and adenosine monophosphate, offering insight into CTCL biomarkers (Le et al., 2018).

#### 2.1.6 NOD SCID mouse in CTCL

The NOD SCID mouse model, formally designated as the NOD. CB17-Prkdc^scid^ strain, is valuable in CTCL research as recipient mice due to its lack of mature T and B cells, making it suitable not only for studying pruritus—a hallmark symptom of CTCL (Prochazka et al., 1992) —but also for investigating tumor growth and early symptoms (Chen et al., 2023; Furutani et al., 2023). Employing the NOD SCID mice for the CTCL model, LW-213 was found to significantly inhibit CTCL tumor growth and enhance survival rates (Yu et al., 2021). It has also facilitated the study of PAK1’s role in CTCL proliferation and the effectiveness of its inhibitors (Wang et al., 2018), and the impact of BIN1 on disease progression via c-FLIP and Fas/FasL-mediated apoptosis (Esmailzadeh et al., 2015). Moreover, treatment combining retinoic acid and histone deacetylase inhibitors has demonstrated antitumor effects (Kato et al., 2016).

#### 2.1.7 CB 17 SCID mouse in CTCL

The CB17 SCID mouse model, originating from the C.B-17 strain, bears a Prkdc^SCID^ gene mutation that results in a profound deficiency in adaptive immunity by impairing T and B lymphocytes (Cattan and Douglas, 1994). This strain, as recipient mouse of CTCL from SS patient-derived skin, provides valuable insights into the pathology and can aid in developing new therapeutic strategies (Charley et al., 1990). Recent research has demonstrated that the combined use of Brentuximab Vedotin (BV) with doxorubicin exhibits significant tumor suppression in the HH cell tumor model in CB17SCID mice, further confirming the potential of this drug combination in the treatment of T-cell lymphomas (Tonozuka et al., 2023).

#### 2.1.8 CB17 SCID beige mouse in CTCL

The CB-17 SCID beige mouse model, due to the combined SCID and beige mutations, possesses an extensive range of immunodeficiencies, including the absence of T cells, B cells, and compromised NK cell function, providing a more comprehensive immunodeficient model than the CB17SCID defect only (Shibata et al., 1997). As a recipient, these mice excel in tumor studies due to their increased tumor growth rates compared to less immunodeficient nude mice, ideal for research on aggressive tumors contrasting slower-progressing SS tumors (Doebbeling, 2010). In CTCL research, these mice, together with the EL4 mouse T-cell lymphoma model, have aided in discovering that the expression of galectin-9 on tumor cells is inversely proportional to CD8^+^ T cell infiltration in the skins of EL4 mouse model and serum levels of galectin-9 correlate with disease severity. Furthermore, high-dose galectin-9 administration demonstrated anti-tumor effects in CTCL, underscore its potential as a therapeutic target (Nakajima et al., 2019).

#### 2.1.9 Rag2 −/− mouse in CTCL

The Rag2−/− mouse model carries a mutation that disables the Rag2 gene, essential for T and B lymphocyte development through V(D)J recombination. This mutation results in a complete absence of mature T and B cells, creating a foundational model for immunodeficiency studies (Hao and Rajewsky, 2001). While not a primary model for CTCL itself, Rag2−/− mice serve as recipients in specific studies, such as those involving subcutaneous injections of modified CD4^+^ T cells from Myc + Cdkn2a −/− mice, to explore the mechanisms of cutaneous hypersensitivity and the immunological roles of IL-7 and IL-15 (Adachi et al., 2015). An enhanced version of this model, the Rag 2−/− γc−/− mice, lack functional T, B, and NK cells due to the knockout of both the Rag2 and the interleukin-2 receptor gamma chain gene (Il2rg or γc), which affects cytokine receptor production. This strain is suitable for xenotransplantation studies on SS, in particular it sustains long-term systemic repopulation with injected SS cell lines or primary cells without immune rejection (van der Fits et al., 2012).

The SRG15 mouse, an advanced version of the Rag 2−/− model, combines *Rag2−/−, γc−/−* mutations and humanized IL15 and human signal regulatory protein alpha (SIRPA) mutations. (Herndler-Brandstetter et al., 2017). These ‘humanized’ mice are engineered to express human IL-15, accommodating the growth of SS tissue samples more effectively than traditional immunodeficient models. Integrating of human IL-15 and SIRPα genes in the SRG15 mice enables them to support human NK and T cells, making them excellent tools for studying human immune cell behaviors (Gao et al., 2023).

### 2.2 Non-immunodeficient mouse models with syngeneic transplantation

Syngeneic transplantation models, wherein syngeneic lymphomas are introduced into the skin of mice, serve as valuable tools for investigating tumor behavior and host-tumor interactions. These models allow for studying tumor dynamics within a genetically consistent background, offering insights into the tumor’s interaction with a native immune system. However, it is crucial to acknowledge that these models have limitations in representing the human immune system and the diverse variants of the disease. Specifically, they lack the complexity and heterogeneity inherent in human CTCLs. Such differences are crucial for researchers to consider, ensuring that the distinct differences between model and human disease are accounted for in research conclusions and clinical applications.

#### 2.2.1 MBL2 mouse model in CTCL

The Mannose-Binding Lectin 2 (MBL2) mouse model, utilizing C57BL/6 mice as a syngeneic platform, creates an auto transplantation model for CTCL by injecting MBL2 lymphoma cells and inducing inflammation with dinitrofluorobenzene (DNFB). Although not based on genuine CTCLs, this model effectively simulates the impact of inflammation observed in CTCL and highlights the potential of anti-inflammatory treatments such as the PARP-1 inhibitor talazoparib and IL-10 suppression in controlling tumor growth (Wu et al., 2011; Wu et al., 2014; Kruglov et al., 2020). Further research has confirmed the efficacy of CD47 blockade agents and CCR2 inhibitors in slowing tumor growth and modulating the tumor microenvironment (Wu et al., 2020; Kruglov et al., 2022). The discovery that rapamycin inhibits tumor growth by highlighting its impact on the metabolism of lymphoma cells, particularly reducing the reliance on aerobic glycolysis, offers a new avenue for metabolic intervention in treating CTCL (Kittipongdaja et al., 2015).

#### 2.2.2 EL4 mouse T-cell lymphoma model

The EL4 mouse model uses a T-cell lymphoma cell line derived from C57BL/6 mice and serves as a syngeneic transplant model for CTCL by virtue of inoculation in the skin with an impact on matching immune system. Studies utilizing this model in CTCL-related research have shown that bexarotene demonstrates immunomodulatory potential by reducing levels of CCL22 (Tanita et al., 2019). Moreover, combining mogamulizumab with PUVA therapy shows enhanced therapeutic effects (Ohuchi et al., 2020). This model has also demonstrated a possible role for CXCL11 in anti-CTCL treatment (Hensbergen et al., 2005), revealed the role of TSLP in promoting a Th2-dominant tumor environment (Takahashi et al., 2016), and identified galectin-9 as a potential new therapeutic target for CTCL (Vieyra-Garcia et al., 2016). Additionally, the EL4 model has elucidated the role of IL9 and its regulatory factors in MF (Vieyra-Garcia et al., 2016), as well as the importance of PlGF in promoting lymphoma cell growth and disease progression in CTCL (Miyagaki et al., 2017).

#### 2.2.3 Murine bone marrow transplantation model

The bone marrow transplantation model for CTCL, examining the *JAK3* ^*A572V*^ mutation, provides insights into lymphocyte development and the mutation’s role in T-cell proliferation and survival. This model reflects the pathological traits of aggressive lymphoproliferative disorders, including CTCL, with manifestations such as skin involvement in human CTCL. Findings indicate the *JAK3* ^*A572V*^ mutation’s capacity to induce a transplantable, diverse CTCL-like disease, exacerbated by trisomy 21, which may result in fatal leukemia from CTCL phenotypes (Cornejo et al., 2009; Rivera-Munoz et al., 2018). Bone marrow transplantation models are relatively complex to operate and require high-standard experimental equipment and environments, which limits the application of the model.

## 3 Non-skin target genetically engineered mouse models (GEMMs) in CTCL research: Carcinogenesis from a to z

Genetically Engineered Mouse Models (GEMMs) offer a physiologically relevant platform to study human CTCL by introducing specific gene modifications (Sharpless and Depinho, 2006). Genomic analysis has identified a number of genes as potential therapeutic targets in CTCL (Bastidas Torres et al., 2018). Given that mice share about 85% genetic similarity with humans (Basheer and Vassiliou, 2021; Mouse Genome Sequencing Consortium et al., 2022), GEMMs facilitate understanding the role of specific genetic modifications in CTCL development. These models enable the study of natural cancer progression and interaction with the immune system from the onset (Dummer et al., 2021). However, non-skin target GEMMs can simulate systemic CTCL pathogenesis but often do not originate from skin-homing CD4^+^ T cells, the main origin of CTCL genesis, limiting their applicability to skin-centric CTCL features.

### 3.1 Knockout mouse models in CTCL research: starting from systemic tumorigenesis

Knockout mouse models are prevalent in CTCL research, enabling the study of gene function by gene deletion, particularly in the core cell type implicated in CTCL, CD4^+^ T cells (Hall et al., 2009). The *CD4CreER* ^*T2*^ transgenic mouse model exemplifies this, where Cre is controlled by the CD4 promoter and gene editing thus selectively targets CD4^+^ T cells. The Cre/lox system used here allows for temporal and cell-specific gene inactivation via tamoxifen-activated CreER^T2^ recombinase. Such inducible knockouts are tools for dissecting gene roles in CD4^+^ T cells, providing insights into their complex functions in immunity and disease progression. Although they may predominantly manifest skin symptoms similar to CTCL, they originate from systemic T cell disorders, aligning more with secondary CTCL types (Aghajani et al., 2012). It highlights the need for careful consideration when extrapolating findings from these models to primary CTCL.

#### 3.1.1 R26STAT3C stopfl/+ CD4Cre mouse model

The *R26STAT3C stopfl/+ CD4Cre* mouse model is utilized to assess the consequences of persistently active STAT3 in CD4^+^ T cells. These mice are genetically engineered to have a modified *STAT3C* gene at the *ROSA26* locus (Casola et al., 2006), which is continuously expressed in CD4 cells due to removing a stop sequence flanked by loxP sites through the CreLoxP system. This persistent activation of STAT3 simulates skin abnormalities akin to those seen in CTCL. Research by Fanok et al. using this model revealed that dysregulated cytokine signaling, particularly aberrations in the IL-2 receptor signaling pathway and the JAK-STAT signaling pathway, as well as imbalances in microenvironmental factors, like the skin microbiome, may promote the onset and progression of CTCL (Fanok et al., 2018).

#### 3.1.2 CD4CreER^T2^Satb1fl/fl Rosa26N1-ICD mouse model

The *CD4CreER* ^*T2*^ *Satb1fl/flRosa26N1-ID* mouse model is designed to study the role of SATB1 protein by Notch1 overexpression (intracellular domain N1-ICD) in CD4^+^ T cells (not only those residing in the skin). This model mirrors advanced - CTCL pathogenesis. *SATB1* loss leads to increased chemokine receptors including CCR4, affecting T-cell migration with the transformation of CD8^+^ T cells into CD4^+^CD8^+^ double-positive T cells and more infiltration of CD3^+^ T cells in the skin of the mice, and CTCL progression. Moreover, with exhibiting CD8 and CD11b co-expression and symptoms like splenomegaly and lymphadenopathy, it is a valuable tool for exploring late-stage CTCL’s advancement and treatment (Harro et al., 2021).

### 3.2 Transgenic mouse models in CTCL research

Transgenic mouse models are created by inserting exogenous DNA into the mouse genome, which allows for precise manipulation of gene expression to assess gene function and its impact during a disease (Viney, 1995). In CTCL research, these models are crucial for exploring genes associated with the disease, providing a window on the mechanisms of CTCL onset and progression.

#### 3.2.1 IL-15 overexpression mouse model

The IL-15 overexpression mouse model uses transgenic technology to introduce an exogenous IL-15 gene into the mouse genome, leading to its overexpression and causing the mice to develop a CTCL-like disease similar to the human condition. IL-15 is a cytokine involved in the maturation of lymphocytes (Fehniger et al., 2001; Schluns and Anthony, 2015). This model mirrors the high levels of IL-15 found in CTCL patients and allows for the observation of clinical symptoms and disease progression *in vivo*, aiding in the understanding of the role of IL-15 in the pathogenesis of CTCL (Mishra et al., 2016; Sindaco et al., 2023). It helps identify potential therapeutic targets, including the regulation of Zeb1 and exploring inhibitors of HDAC and miR-214 (Mishra et al., 2016; Kohnken et al., 2019). The highlighted negative regulatory relationship between miR-29b and BRD4 opens up new avenues for preventing the progression of CTCL (Kohnken et al., 2018).

It is important to note that while the IL-15 overexpression mouse model provides valuable insights into the role of IL-15 in CTCL, it cannot explain the mechanisms by which IL-15 overexpression occurs in patients. Therefore, further studies are needed to understand this fully and develop effective treatments.

## 4 The next top mouse models: Genetically engineered mouse models (GEMMs) advancing CTCL research by targeting skin-homing CD4^+^ T Cells in combination with inflammation

Recent advancements in CTCL research have led to the creation of ‘autochthonous mouse models’, effectively replicating the disease’s initial skin-based progression (Bresin et al., 2022). These models, designed to modify genes in skin-homing CD4^+^ T cells along with localized inflammation, closely emulate CTCL’s natural development (Luo et al., 2023). They have markedly enhanced our understanding of early-stage CTCL genesis and show promise in designing early interventions. However, these models primarily reflect very early-stage premalignant traits of CTCL, as yet lacking progression to the more aggressive malignant features of advanced CTCL. This gap highlights the need for continued development and refinement of these models to fully represent the disease’s advanced stages.

### 4.1 Socs1 flox CD4CreER^T2^ mouse model

The *Socs1 flox CD4CreER* ^*T2*^ mouse model is important in CTCL research, especially for understanding the role of *SOCS1* in the progression of the disease. *SOCS1* has been found to be deficient in patients with CTCL, particularly in those with early-stage MF. By selectively deleting the *Socs1* gene in the CD4^+^ T cells of mice—which is accomplished by the cross breeding of Cre transgenic and *Socs1* floxed mice—researchers can observe the effects of the absence of this gene in skin-resident T cells involved in inflammation of the skin (activation of Cre by topical application of tamoxifen on the inflamed skin). This model has revealed that isolated loss of the *Socs1* gene does not prompt early-stage MF, even when coupled with short term inflammatory challenges. However, with protracted challenges - specifically, over a period of 20 weeks—the skin inflammation becomes autonomous and mirrors early-stage MF with lymphoproliferation, offering a more profound understanding of the disease’s early development and potential intervention (Luo et al., 2022; Luo et al., 2023).

The next step will be the analogous experiments involving the Hnrnpk flox model as a key target in the JAK-STAT3 pathway for CTCL. which can be combined with a defect in *CDKN2A* to enhance tumor development. *Hnrnpk flox CD4CreER* ^*T2*^ mouse model can be utilized to examine the impact of *HNRNPK* gene haplodeficiency or its complete absence in CD4^+^ T cells, aiming to elucidate its role in CTCL pathogenesis (Bastidas Torres et al., 2018; Park et al., 2021). This approach is informed by previous studies indicating significant deletions of *Hnrnpk* in the JAK-STAT pathway among CTCL patients. This builds on the foundational research conducted using the *Socs1 flox CD4CreER* ^*T2*^ mouse model, which has paved the way for exploring the effects of these genes related to CTCL, both individually and in combination.

## 5 Conclusion

Balancing the strengths and weaknesses of *in vivo* models of CTCL and continuously refining them is essential. *In vivo* mouse models, particularly through transplantation systems and GEMMs, have significantly enhanced our understanding of CTCL, a unique non-Hodgkin lymphoma. However, these models carry inherent limitations. Xenotransplant models, while efficient and established, offer insights into tumor progression and treatment responses but miss the full immune interactions present in humans. GEMM models, ideal for studying genetic factors, may not completely replicate the skin-origin nature of CTCL, posing challenges in representing early-stage CTCL pathogenesis.

The skin-targeted GEMMs, though still lacking complete evidence of malignant progression, closely mimic CTCL’s origin and early changes. Future research should focus on leveraging emerging technologies like CRISPR-Cas9 gene editing to enhance the accuracy and relevance of these models. By introducing selectively modified CTCL-related genes in skin-homing CD4+T cells with inflammation, the next top mouse models pave the way for exploring gene effects and understanding the genesis of early-stage CTCL. This understanding is key to developing early and effective interventions.
